# Prevalencia de la comorbilidad tuberculosis y diabetes mellitus en Paraguay, 2016 y 2017

**DOI:** 10.26633/RPSP.2019.105

**Published:** 2019-12-20

**Authors:** Cynthia Céspedes, Lucelly López, Sarita Aguirre, Alberto Mendoza-Ticona

**Affiliations:** 1 Programa Nacional de Control de la Tuberculosis Ministerio de Salud Pública y Bienestar Social Asunción Paraguay Programa Nacional de Control de la Tuberculosis, Ministerio de Salud Pública y Bienestar Social, Asunción, Paraguay.; 2 Universidad Pontificia Bolivariana Medellín Colombia Universidad Pontificia Bolivariana, Medellín, Colombia.; 3 Universidad Nacional de San Agustín Arequipa Perú Universidad Nacional de San Agustín, Arequipa, Perú.

**Keywords:** Tuberculosis, diabetes mellitus, comorbilidad, Paraguay, Tuberculosis, diabetes mellitus, comorbidity, Paraguay, Tuberculose, diabetes mellitus, comorbidade, Paraguai

## Abstract

**Objetivo.:**

Estimar la prevalencia nacional y regional de la comorbilidad tuberculosis (TB) y diabetes mellitus (DM) e identificar los factores asociados con esta comorbilidad en Paraguay.

**Métodos.:**

Estudio transversal en pacientes con TB notificada en 2016 y 2017 y registrados en la base de datos del Programa Nacional de Control de la TB. La prevalencia de DM, definida por autonotificación, se estimó en pacientes con TB. Para conocer los factores asociados con la comorbilidad TB-DM se empleó un modelo multivariante de regresión binomial para ajustar las razones de prevalencia (RP) según los errores estándar por el clúster de región sanitaria.

**Resultados.:**

Entre 2016 y 2017 se notificaron 5 315 casos de TB. La prevalencia de la comorbilidad TB-DM fue 6,3% en 2016, 6,0% en 2017 y 6,2% en ambos años. Fue más alta en Itapúa (9,2%), Alto Paraguay (8,0%), Alto Paraná (7,5%), Central (7,4%) y Asunción (7,2%). La mediana de edad de personas con DM fue más alta que la de las que no tenían DM (55 y 33 años; P < 0,001). Tener una edad mayor de 45 años (RP = 18,3), el sexo femenino, antecedente de hipertensión arterial (HTA) (RP = 2,17), baciloscopía de diagnóstico de tres cruces (RP 1,98), y antecedente de enfermedad pulmonar obstructiva crónica (EPOC) (RP 1,68) estuvieron asociados con mayor comorbilidad. En cambio, se asociaron con menor comorbilidad pertenecer a la población indígena (RP = 0,26), la infección por el virus de la inmunodeficiencia humana (RP = 0,44), historia de adicción a drogas (RP = 0,49), el sexo masculino (RP = 0,64), y la TB extrapulmonar (RP = 0,75).

**Conclusiones.:**

La prevalencia de la comorbilidad de TB y DM en Paraguay, por autonotificación, fue 6,2% en el periodo 2016-2017 y varió entre las regiones sanitarias. La edad, una alta carga bacilar al diagnóstico y la comorbilidad con HTA y EPOC se asociaron a una mayor comorbilidad. Estos hallazgos permitirán priorizar grupos de población para aumentar rendimiento del cribado, diagnóstico, tratamiento y prevención de la comorbilidad TB-DM en Paraguay.

## Abstract (en)

**Objective.:**

To estimate the national and regional prevalence of tuberculosis (TB) and diabetes mellitus (DM) co-morbidity and identify the factors associated with this co-morbidity in Paraguay.

**Methods.:**

Cross-sectional study in patients with TB notified in 2016 and 2017 and registered in the database of the National TB Control Program. The prevalence of self-reported DM was estimated in patients with TB. A multivariate binomial regression model was used to know the factors associated with TB-DM co-morbidity to adjust the prevalence ratios (PR) according to standard errors by health region.

**Results.:**

Between 2016 and 2017, 5 315 cases of TB were reported. The prevalence of TB-DM co-morbidity was 6.3% in 2016, 6.0% in 2017, and 6.2% in both years. It was highest in Itapua (9.2%), Alto Paraguay (8.0%), Alto Parana (7.5%), Central (7.4%) and Asuncion (7.2%). The median age of people with DM was higher than that of those without DM (55 vs 33 years; P < 0.001). Being older than 45 years (RP = 18.3), history of hypertension (RP = 2.17), diagnostic baciloscopy +++ (RP 1.98), and history of chronic obstructive pulmonary disease (COPD) (RP 1.68) were associated with greater co-morbidity. A lower co-morbidity was associated with belonging to the indigenous population (RP = 0.26), human immunodeficiency virus infection (RP = 0.44), history of drug dependence (RP = 0.49), male sex (RP = 0.64), and extrapulmonary TB (RP = 0.75).

**Conclusions.:**

The prevalence of self-reported co-morbidity of TB-DM in Paraguay was 6.2% in 2016-2017 and varied between health regions. Age, sex, high bacillary burden at diagnosis and co-morbidity with hypertension and COPD were associated with higher co-morbidity. These findings will allow prioritizing population groups to increase screening performance, diagnosis, treatment and prevention of TB-DM co-morbidity in Paraguay.

## RESUMO

**Objetivo.:**

Estimar a prevalência nacional e regional de comorbidade entre tuberculose (TB) e diabetes mellitus (DM) no Paraguai e identificar os fatores associados a essa comorbidade.

**Métodos.:**

Estudo transversal em pacientes com TB notificados em 2016 e 2017 e cadastrados na base de dados do Programa Nacional de Controle da TB. A prevalência de DM, definida por autorrelato, foi estimada em pacientes com TB. Para conhecer os fatores associados à comorbidade TB-DM, foi utilizado um modelo de regressão binomial multivariada para ajustar as razões de prevalência (RP) de acordo com os erros padrão do cluster da região de saúde.

**Resultados.:**

Em 2016 e 2017 foram notificados 5 315 casos de TB. A prevalência de comorbidade TB-DM foi de 6,3% em 2016, 6,0% em 2017 e 6,2% para o período dos 2 anos. As prevalências mais altas foram observadas em Itapúa (9,2%), Alto Paraguai (8,0%), Alto Paraná (7,5%), Central (7,4%) e Assunção (7,2%). A mediana de idade foi mais alta em pessoas com DM do que naquelas sem DM (55 e 33 anos; *P* <0,001). Ter idade superior a 45 anos (RP = 18,3), história de hipertensão arterial (HAS) (RP = 2,17), baciloscopia diagnóstica (+++) (RP = 1,98) e história de doença pulmonar obstrutiva crônica (DPOC) (RP = 1,68) foram associados a maior comorbidade. Por sua vez, pertencer à população indígena (RP = 0,26), infecção pelo vírus da imunodeficiência humana (RP = 0,44), histórico de dependência de drogas (RP = 0,49), sexo masculino (RP = 0,64) e TB extrapulmonar (RP = 0,75) estiveram associados a menor comorbidade.

**Conclusões.:**

A prevalência de comorbidade TB-DM no Paraguai, determinada a partir de autorrelato, foi de 6,2% no período 2016-2017 e variou entre as regiões de saúde. Idade, sexo, alta carga bacilar no diagnóstico e comorbidade com HAS e DPOC foram fatores associados a maior comorbidade. Esses achados permitirão priorizar grupos populacionais para aumentar o desempenho da triagem, diagnóstico, tratamento e prevenção da comorbidade TB-DM no Paraguai.

La tuberculosis (TB) continúa siendo un problema de salud pública mundial, tanto por su elevada carga de enfermedad como por sus graves consecuencias sociales y económicas, especialmente en países de ingresos medianos y bajos (1). En los últimos años, y como consecuencia de cambios transicionales demográficos, epidemiológicos y de los hábitos alimenticios, ha emergido la pandemia de la diabetes mellitus (DM) (2). La globalización de estilos de vida no saludables y de otros determinantes sociales de la salud ha resultado en un aumento de la carga mundial de DM en la población adulta de 108 millones de casos en 1980 a 382 millones 2013, y se espera que este número se eleve a 592 millones en 2035 (3). Se reconoce que la DM triplica el riesgo de TB activa y empeora el resultado de su tratamiento, retrasa la conversión bacteriológica y se relaciona con una mayor proporción de fracasos y recaídas (4, 5). A pesar de esta conocida relación, los datos a escala global, regional y nacional sobre esta comorbilidad son limitados. Se carece de cifras de notificaciones o estimaciones sobre la real carga de la comorbilidad TB-DM y sus consecuencias clínicas en los informes globales o regionales sobre la TB (6).

En Paraguay, tanto la TB como la DM son problemas de salud pública. En 2017, la prevalencia estimada de DM en la población fue de casi 6,5%, más alta en las mujeres y en los mayores de 60 años (7). Sin embargo, en el estudio AsuRiesgo de Paraguay de 2015 se notificó una prevalencia más elevada de DM en la población general mayor de 18 años de edad (13,3%) (8). Por su parte, el sistema de información del Programa Nacional de Control de la Tuberculosis (PNCT) notificó en 2016 un total de 2 436 casos de TB que corresponden a una incidencia anual de 35,6 casos por 100 000 habitantes. El 85% de ellos corresponden a casos nuevos de TB y 68% se confirman bacteriológicamente (9). La autonotificación de la DM por todo paciente recientemente diagnosticado de TB se implementó desde 2016; no obstante, la información sobre la comorbilidad de la TB-DM recolectada aún no se ha descrito ni analizado.

El presente estudio tuvo por objetivo estimar la prevalencia de la comorbilidad TB-DM a nivel nacional y por zonas sanitarias de Paraguay, así como identificar los factores asociados con esta comorbilidad y comparar las principales características clínicoepidemiológicas de los pacientes que padecen TB con y sin DM.

## MATERIALES Y MÉTODOS

Se realizó una investigación operativa de tipo observacional, con un diseño transversal, utilizando la base de datos del PNCT de Paraguay de 2016 y 2017. El registro se realiza a través del Sistema de Información Experto del PNCT del Ministerio de Salud (10).

Paraguay está clasificado como país de ingreso medio alto por el Banco Mundial desde el 2018. Cuenta con una población aproximada de 7,1 millones de habitantes, distribuidos en 18 regiones sanitarias. El PNCT es el ente programático y normativo responsable del control de la TB en el país. Todos los servicios de salud del Ministerio de Salud Pública y Bienestar Social y de la Seguridad Social notifican los casos al PNCT. El diagnóstico y el tratamiento de la TB en Paraguay son gratuitos.

En el estudio se incluyeron todos los pacientes con todas las formas de TB, que habían autonotificado padecer o no DM (dato obligatorio en la ficha del programa desde 2016) y estaban registrados en la base oficial del PNCT. Se excluyeron aquellos con registros duplicados en los dos años de estudio y los casos de TB resistente a medicamentos.

Las variables utilizadas en este estudio fueron las siguientes: región sanitaria, año de estudio, autonotificación de la DM, edad, sexo, pertenecer a la población indígena, coinfección por el VIH, otras comorbilidades (hipertensión arterial, EPOC, adicción a psicofármacos, alcoholismo), baciloscopía de diagnóstico, localización de la enfermedad. La información se exportó, almacenó y tabulo en el programa Excel (Microsoft, Estados Unidos de América).

Para estimar la prevalencia de DM en los casos notificados de TB y tratados en 2016 y 2017 a nivel nacional y regional se usó como numerador el número de casos de morbilidad TB-DM y como denominador, el total de casos de TB notificados en el mismo año. Se compararon las características epidemiológicas y clínicas de los pacientes con y sin comorbilidad TB-DM. Los porcentajes se compararon con la prueba de Chi-cuadrado. Para conocer los factores asociados con la comorbilidad TB-DM se construyó un modelo multivariante binomial a fin de ajustar las razones de prevalencia (RP) según los errores estándar por el clúster de región sanitaria. Se aceptó un nivel de significación estadística de 0,05. Se trabajó con el programa IBM SPSS 24 (IBM Company, Estados Unidos de América).

El protocolo fue revisado y aprobado por el Comité de Ética de la Organización Panamericana de la Salud (OPS) y por el Comité Institucional de Ética del Laboratorio Central de Salud Pública de Paraguay. Se garantizó la confidencialidad de los participantes en todos los momentos del desarrollo de la investigación. Se emplearon bases de datos de investigación sin ningún dato que permitiera identificarlos. Las bases contaron con clave de acceso solo disponible para los investigadores.

## RESULTADOS

Durante 2016 y 2017 se notificaron 5 573 casos de TB en Paraguay. Se excluyeron 438 casos: 428 por duplicidad de registro y 10 por tener TB resistente a medicamentos. Ingresaron en el estudio 5 135 casos, que representan el 92% del total de los casos notificados.

**FIGURA 1.**
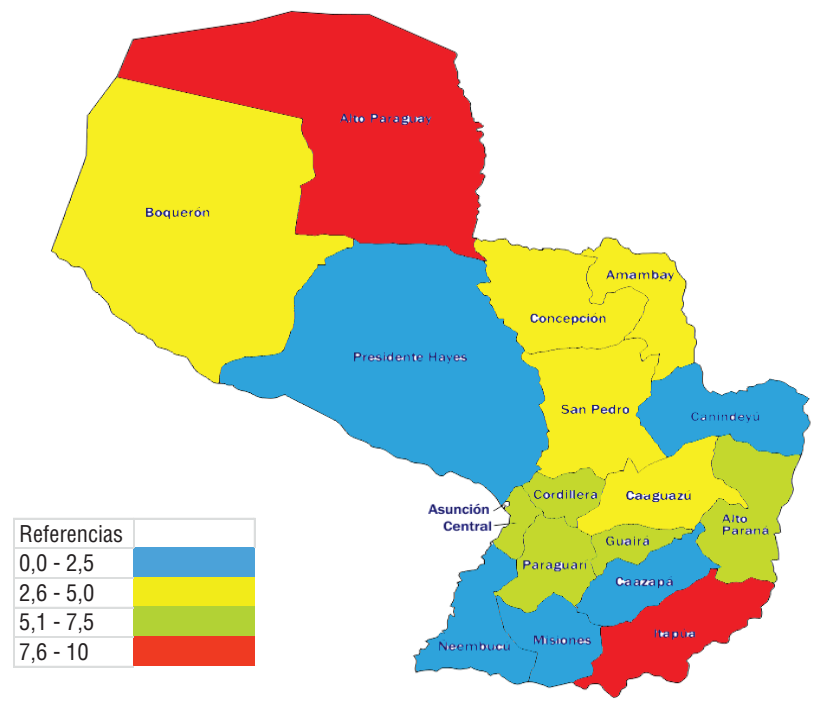
Prevalencia de diabetes mellitus autonotificada por pacientes con tuberculosis por región sanitaria, Paraguay, 2016–2017^1^

De los 5 135 casos de TB, 317 notificaron tener DM, con una prevalencia global de 6,2% (IC95%: 5,2%-7,2%). En 2016 la prevalencia fue de 152 en 2412 casos (6,3%) y en 2017, de 165 en 2723 (6,0%). En el cuadro 1 y en la figura 1 se muestran los resultados de prevalencia de DM en pacientes con TB por región sanitaria para 2016 y 2017. El rango de prevalencia en las regiones sanitarias fue de 0 a 9,2%. Las regiones sanitarias con prevalencias por encima de la media nacional fueron Itapúa, Alto Paraguay, Alto Paraná, Central y Asunción y las regiones sin casos de TB-DM Caazapá, Misiones, Ñeembucú, Canindeyu y Presidente Hayes.

**CUADRO 1.** Prevalencia de diabetes mellitus autonotificada por pacientes con tuberculosis por región sanitaria y año, Paraguay, 2016-2017

Región sanitaria	Año		Total
	2016	2017	n/N (%)
	n/N (%)	n/N (%)	
Itapúa	12/153 (7,8)	15/142 (10,6)	27/295 (9,2)
Alto Paraguay	1/12 (8,3)	1/13 (7,7)	2/25 (8,0)
Alto Paraná	26/331 (7,9)	29/401 (7,2)	55/732 (7,5)
Central	27/300 (9,0)	21/352 (6,0)	48/652 (7,4)
Asunción	63/896 (7,0)	74/1004 (7,4)	137/1900 (7,2)
Paraguarí	3/44 (6,8)	2/37 (5,4)	5/81 (6,2)
Cordillera	6/69 (8,7)	2/74 (2,7)	8/143 (5,6)
Guairá	2/46 (4,3)	2/31 (6,5)	4/77 (5,2)
Boquerón	3/71 (4,2)	3/63 (4,8)	6/134 (4,5)
Concepción	2/66 (3,0)	4/76 (5,3)	6/142 (4,2)
San Pedro Sur	0/31 (0)	2/26 (7,7)	2/57 (3,5)
San Pedro Norte	1/41 (2,4)	2/51 (3,9)	3/92 (3,3)
Amambay	3/79 (3,8)	2/73 (2,7)	5/152 (3,3)
Caaguazú	3/117 (2,6)	6/192 (3,1)	9/309 (2,9)
Misiones	0/23 (0)	0/28 (0)	0/51 (0)
Ñeembucú	0/9 (0)	0/10 (0)	0/19 (0)
Canindeyu	0/26 (0)	0/40 (0)	0/66 (0)
Presidente Hayes	0/71 (0)	0/81 (0)	0/152 (0)
Caazapá	0/16 (0)	0/20 (0)	0/36 (0)
Extranjeros	0/11 (0)	0/9 (0)	0/20 (0)
Total del país	152/2412 (6,3)	165/2723 (6,0)	317/5315 (6,2)

*Fuente:* elaboración propia a partir de los resultados presentados en este estudio.

*Fuente*: elaboración propia a partir de los resultados presentados en este estudio.

^1^ Las denominaciones empleadas y la forma como aparecen presentados los datos en el mapa no implican, por parte de la RPSP ni de la OPS, juicio alguno sobre la condición jurídica de países, territorios, ciudades o zonas ni respecto del trazado de sus fronteras o límites.

La mediana de edad de las personas con DM fue mayor que la de las personas sin DM, 55 (23 – 64) y 33 (23 – 50) (*p* < 0,001), respectivamente. El 36,9% de las personas con DM fueron mujeres. En cambio, la proporción de mujeres fue menor en el grupo sin DM (29,6%) (p = 0,002). En el cuadro 2 se comparan las características clínicas y epidemiológicas de los pacientes con TB con y sin DM. Como es de esperar, la prevalencia de DM es más alta en las personas mayores de 45 años, por lo que distribución por grupos de edad fue diferente entre las personas con y sin DM. También se encontró una mayor prevalencia de la comorbilidad TB-DM en personas que padecían enfermedad pulmonar obstructiva crónica (EPOC) o hipertensión arterial (HTA) y en el informe de la baciloscopía de diagnóstico. En cambio, en la población indígena, las personas que viven con el VIH y las que padecen adicción a psicofármacos la prevalencia de DM fue menor. Ésta fue más elevada en mujeres que en hombres, aunque la diferencia no fue estadísticamente significativa en el análisis bivariante. Tampoco hubo diferencias en la localización de la TB (pulmonar o extrapulmonar) entre pacientes con y sin historia de tratamiento anti-TB previo, en pacientes privados o no de libertad o en consumidores de alcohol o abstemios (cuadro 2).

Se investigaron los factores asociados con la comorbilidad TB-DM en personas con TB. En el cuadro 3 aparecen las RP brutas y ajustadas. Los factores asociados con el aumento de la comorbilidad TB-DM en Paraguay según el modelo ajustado fueron la edad mayor a 45 años, el sexo femenino, tener un resultado de baciloscopía de diagnóstico de dos y tres cruces, y tener comorbilidad con EPOC y HTA. En cambio, los factores asociados con menor prevalencia de comorbilidad TB-DM fueron pertenecer a la población indígena, tener TB extrapulmonar, padecer adicción a psicofármacos y coinfección por el VIH.

**CUADRO 2.** Características clínicas y epidemiológicas de los pacientes con tuberculosis, con y sin diabetes mellitus, Paraguay, 2016-2017

Características	Con DM	Sin DM	Total	p
	N = 317	N = 4 818	N = 5 135	
	n (%)	n (%)	n (%)	
Grupo de edad (años)				< 0,001
< 15	2 (0,5)	423 (99,5)	425 (8,3)	
15–30	15 (3,0)	1 726 (99,1)	1741 (33,9)	
31–45	65 (5,0)	1 224 (95,0)	1289 (25,1)	
46–60	118 (13,7)	745 (86,3)	863 (16,8)	
> 61	117 (14,5)	692 (85,5)	809 (15,8)	
Sexo				0,22
Masculino	204 (5,7)	3 394 (94,3)	3 598 (70,1)	
Femenino	113 (7,4)	1 424 (92,6)	1 537 (29,9)	
Población indígena				< 0,001
Sí	14 (1,6)	848 (98,4)	862 (16,8)	
No	303 (7,1)	3 970 (92,9)	4 273 (83,2)	
Infección por VIH				< 0,001
Sí	9 (2,1)	411 (97,9)	420 (8,2)	
No	308 (6,5)	4 407 (93,5)	4 715 (91,8)	
EPOC				< 0,001
Sí	23 (15,8)	123 (84,2)	420 (8,2)	
No	294 (5,9)	4 695 (94,1)	4 989 (97,2)	
HTA				< 0,001
Sí	9 (30,0)	21 (70,0)	30 (0,6)	
No	308 (6,0)	4 797 (94,0)	5 105 (99,4)	
Privado de libertad				0,225
Sí	31 (5,1)	581 (94,9)	612(11,9)	
No	286 (6,3)	4 237 (93,7)	4 523 (88,1)	
Adicción a psicofármacos				<0,001
Sí	11 (1,7)	634 (98,3)	645 (12,6)	
No	306 (6,8)	4 184 (93,2)	4 490 (87,4)	
Consumo de alcohol				0,43
Sí	29 (7,1)	381 (92,9)	410 (8,0)	
No	288 (6,1)	4 437 (93,9)	4 725 (92,0)	
Tratamiento previo				0,198
Caso nuevo	237 (6,7)	3 326 (93,3)	3 563 (69,4)	
Tratamiento	0 (0)	18 (100)	18 (0,4)	
después del fracaso			
Recaída	10 (4,8)	200 (95,2)	210 (4,1)	
Tratamiento postPS	13 (6,3)	192 (93,7)	205 (4,0)	
Sin datos	57 (5,0)	1 082 (95,0)	1 139 (22,2)	
Localización de la TB				0,474
Pulmonar	278 (6,3)	4 122 (93,7)	4 400 (85,7)	
Extrapulmonar	24 (4,9)	464 (95,1)	488 (9,5)	
Ambas	15 (6,1)	232 (93,9)	247 (4,8)	
Baciloscopía de diagnóstico				< 0,001
Negativa	46 (4,5)	979 (95,5)	1 025 (20,0)	
+	58 (6,3)	866 (93,7)	924 (18,0)	
++	52 (7,3)	664 (92,7)	716 (13,9)	
+++	108 (9,2)	1 060 (90,8)	1 168 (22,7)	
Paucibacilar	53 (4,1)	1 249 (95,9)	1 302 (25,4)	

*Fuente*: elaboración propia a partir de los resultados presentados en este estudio.

DM: diabetes mellitus; VIH: virus de la inmunodeficiencia humana; EPOC: enfermedad pulmonar obstructiva crónica; HTA: hipertensión arterial; TB: tuberculosis; PS: pérdida de seguimiento.

**CUADRO 3.** Factores asociados con la presencia de diabetes mellitus en pacientes con tuberculosis, Paraguay, 2016-2017

Factores	RP bruta (IC95%)	p	RP ajustada (IC95%)^1^	*p*^2^
Grupo de edad (añós)				
16–45	5,61 (1,04-30,1)	0,044	4,13 (0,76-22,6)	0,101
≥ 46	29,9 (5,69-156,6)	< 0,001	18,9 (3,65-98,2)	< 0,001
Sexo (masculino)	0,77 (0,64 0,92)	0,006	0,65 (0,51-0,83)	0,001
Baciloscopía de diagnóstico^3^				
+	1,39 (1,06-1,84)	0,017	1,19 (0,88-1,61)	0,263
++	1,61 (1,09-2,39)	0,016	1,58 (1,13-2,22)	0,008
+++	2,06 (1,64-2,58)	< 0,001	1,88 (1,56-2,428)	< 0,001
Paucibacilar	0,90 (0,73-1,11)	0,36	0,98 (0,79-1,22)	0,879
Población indígena	0,22 (0,10-0,48)	0,001	0,25 (0,13-0,49)	< 0,001
TB extrapulmonar	0,78 (0,60-1,01)	0,063	0,99 (0,78-1,26)	0,926
Adicciones	0,25 (0,12-0,49)	< 0,001	0,48 (0,26-0,88)	0,018
VIH	0,32 (0,23-0,46)	< 0,001	0,44 (0,35-0,59)	< 0,001
EPOC	2,67 (2,24-3,17)	< 0,001	1,63 (1,328-2,08)	< 0,001
HTA	4,97 (2,77-8,91)	< 0,001	2,19 (1,18-4,08)	0,013
Privados de libertad	0,80 (0,61-1,05)	0,109	0,80 (0,65-0,98)	0,032

*Fuente*: elaboración propia a partir de los resultados presentados en este estudio.

RP: razón de prevalencias; IC95%: intervalo de confianza del 95%; VIH: virus de la inmunodeficiencia humana; EPOC: enfermedad pulmonar obstructiva crónica; HTA: hipertensión arterial.

^1^Los errores estándar de los IC fueron ajustados por la región sanitaria de diagnóstico.

^2^Los valores *p* proceden del modelo de regresión binomial; en el caso de los brutos, de regresiones binomiales simples con ajuste de errores por región sanitaria y en el ajustado, del modelo multivariante.

^3^El grupo de referencia de la baciloscopía de diagnóstico es 0 cruces.

## DISCUSIÓN

Este estudio constituye, según el conocimiento de los autores, el primer informe sobre la prevalencia de la comorbilidad TB-DM en Paraguay de ámbito nacional. El 6,2% notificado de DM en pacientes con TB es similar a la prevalencia de DM de la población general, conforme a los informes del Programa de Diabetes del Paraguay (7), pero menor al 13,3% notificado en el estudio AsuRiesgo (8). Sin embargo, se debe resaltar que la prevalencia del estudio AsuRiesgo se obtuvo de una cohorte seleccionada en un solo centro, el Hospital Central de Instituto de Previsión Social en Asunción, donde la población indígena no suele ser atendida. El 6,2% de la prevalencia de la comorbilidad TB-DM consistiría en una parte del total de la prevalencia real, puesto que se basa en la autonotificación de la DM, por lo que a estas cifras estimadas se deberían añadir los casos con DM que se diagnostican en el momento de diagnosticar la TB. Asimismo, se notifican la prevalencia de las 18 regiones sanitarias del país y los factores asociados con esta comorbilidad, teniendo en cuenta la información recolectada de manera programática por el PNCT.

La prevalencia estimada de comorbilidad en este estudio se basa en información obtenida por autonotificación y es menor que la notificada en estudios que han evaluado progresivamente esta comorbilidad y utilizado métodos de diagnóstico de DM en todos los pacientes con TB al diagnosticarlos, como el estudio multicéntrico TANDEM (11). En ese estudio las prevalencias estimadas en Indonesia, Perú, Rumania y Sudáfrica fueron, respectivamente, 19,7, 12,3, 12,3 y 10,9%. Los autores indicaron que dos tercios de los pacientes con DM ya tenían un diagnóstico previo de DM y el diagnóstico de DM del tercio restante se determinó al diagnosticarles la TB (11). Extrapolando este factor a nuestros resultados, se estima una prevalencia de 9,3% para Paraguay, considerando que 6,2% corresponde a dos tercios de la prevalencia real. Este valor se aproxima al del informe de Perú y está por debajo de los de México, que ascienden hasta 36% (12).

Es importante destacar que la prevalencia estimada de 9,3% de la comorbilidad TB-DM en Paraguay estaría por encima del 8% de la prevalencia de la coinfección TB-VIH que el país notificó a la OMS en 2017 (13). Desde 2018, se viene implementando en el país el cribado de la DM con glucosa en ayunas, por lo que se espera tener datos programáticos más exactos de esta comorbilidad. Las regiones sanitarias con mayor prevalencia de la comorbilidad TB-DM (Asunción, Itapúa, Alto Paraná, Central y Alto Paraguay) tienen la peculiaridad de ser mayormente de población urbana con un cinturón de área marginal y en ellas también se notifica la mayor prevalencia de DM de la población general de todo el país (7). En cambio, las zonas con menor prevalencia de DM son mayormente rurales y tienen una proporción alta de población indígena.

La mediana de edad de los pacientes con TB-DM fue más alta que la mediana de la edad en pacientes sin DM. La DM fue más frecuente en personas con TB mayores de 45 años en Paraguay, lo que coincide con el estudio TANDEM, donde la mediana de edad de los pacientes con DM y TB fue de 55 años frente a 33 que tenían TB sin DM (11). Además, en una revisión sistemática sobre la prevalencia y los factores asociados con la TB y la DM se encontró una asociación entre TB-DM y las edades avanzadas (14). En nuestro estudio, el sexo femenino, una baciloscopía de dos y tres cruces, y otras enfermedades crónicas, como la HTA y la EPOC, están directamente asociadas con una mayor prevalencia de DM en los pacientes con TB en Paraguay. En cambio, la coinfección por el VIH, tener TB extrapulmonar, pertenecer a poblaciones indígenas y tener adicciones a psicofármacos se asoció con menor frecuencia de TB-DM. El mayor riesgo en mujeres no se detectó en el estudio TANDEM (11) y la revisión sistemática mencionada incluye estudios que muestran riesgo aumentado en personas de ambos sexos (14).

Otros factores asociados con la comorbilidad TB-DM señalados en la bibliografía son residir en una zona urbana, tabaquismo, sedentarismo, pobre control de la glicemia y tener historia familiar de DM (14, 15). Muchos de estos factores no se han podido evaluar en el presente estudio por su diseño retrospectivo. Otro hallazgo importante del presente estudio es que la comorbilidad TB-DM se asocia con mayor prevalencia de otras enfermedades crónicas, como la HTA y la EPOC. A medida que la población envejece, la prevalencia de enfermedades crónicas aumenta. En una reciente experiencia en Angola, que integró los servicios de TB y de enfermedades no comunicables, se comprobó que los pacientes con TB tienen una comorbilidad considerable con HTA y DM (19,4% y 6,3%, respectivamente). No obstante, a diferencia de nuestros resultados, la prevalencia de TB-DM notificada por este grupo fue mayor en hombres que en mujeres. (16). Los resultados presentados refuerzan la necesidad de garantizar el cribado de la DM y de la HTA en los servicios de TB, sobre todo en personas mayores de 45 años. La integración de los servicios de salud tanto para enfermedades comunicables como para no comunicables que constituyen problemas de salud pública es una política que debe implementarse en Paraguay.

La DM condiciona formas más graves de TB, como, por ejemplo, tener baciloscopías de diagnóstico con mayor carga de bacilos, como se ha encontrado en el presente estudio. Esto condiciona una demora en la conversión de la baciloscopía y el cultivo y una mayor proporción de respuestas pobres al tratamiento con más fracasos y recaídas (17, 18).

La población indígena se asocia indirectamente con la presencia de la comorbilidad TB-DM, lo que puede deberse a que en esta población los estilos de vida y hábitos alimenticios se conservan en el tiempo y la DM es menos prevalente que en las poblaciones mestizas de América Latina. En el estudio poblacional en Paraguay también se ha encontrado una prevalencia de DM baja en esta población (7). Ello también puede responder a las barreras de acceso a los servicios de diagnóstico de enfermedades crónicas, lo cual debe evaluarse prospectivamente con intervenciones para mejorar el acceso de estas poblaciones a los servicios de salud (19).

Este estudio tiene varias limitaciones. La principal es que la estimación de la prevalencia se basa en la autonotificación obligatoria de la DM de pacientes con TB como único método de diagnóstico implementado en los registros de TB desde 2016. A partir de 2018, el PNCT está introduciendo el cribado obligatorio con la prueba de glicemia en ayunas. Este primer informe sirve como referencia para conocer el cambio que se vaya produciendo en esta intervención sanitaria. Otra limitación es no haber realizado el análisis de variables clínicas y epidemiológicas importantes que no se registran de manera programática (el índice de masa corporal, los antecedentes familiares de DM, el nivel socioeconómico, las manifestaciones radiológicas de la TB, el resultado del tratamiento de la TB o la información sobre el tratamiento y el control de la DM).

En conclusión, la prevalencia de DM autonotificada por pacientes con TB es alta en Paraguay. Su media nacional es 6,2%, oscila de 0 a 9,2% entre regiones, sanitarias y alcanza el máximo en Itapúa, Alto Paraguay, Alto Paraná, Central y Asunción. Es muy probable que estas prevalencias asciendan con la implementación del cribado activo de la DM en todos los casos de TB, y supere el 8% de la coinfección TB-VIH notificada en Paraguay. Además, los factores asociados con mayor prevalencia de TB-DM son el ser mayor de 45 años, el sexo femenino, una baciloscopía de diagnóstico de dos y tres cruces, y tener comorbilidad con HTA y EPOC. Por su parte, pertenecer a la población indígena, tener la infección por el VIH, las formas extrapulmonares de la TB y la adicción a psicofármacos se asociaron con una menor prevalencia de comorbilidad TB-DM. Los resultados de este estudio han sido presentados a la administración del PNCT de Paraguay para sustentar la priorización de intervenciones de cribado, prevención, diagnóstico y tratamiento oportuno de esta comorbilidad en todo el país

## Contribución de los autores.

CC y SA concibieron el estudio original. CC y LL recolectaron y organizaron los datos de estudio, LL y AM los analizaron, y todos juntos los interpretaron. CC y AM escribieron el manuscrito. Todos los autores revisaron y aprobaron la versión final.

## Agradecimientos.

Esta investigación se llevó a cabo mediante la iniciativa de capacitación estructurada en investigación operativa (SORT IT, por su siglas en inglés), una alianza mundial dirigida por el Programa Especial de Investigación y Capacitación de Enfermedades Tropicales de la Organización Mundial de la Salud (OMS/TDR) y el Departamento de Enfermedades Transmisibles y Determinantes Ambientales de la Organización Panamericana de la Salud (OPS).

## Financiación.

Se obtuvo financiamiento de la Oficina Regional de la OPS. Los financiadores no desempeñaron ningún papel en el diseño del estudio, la recopilación y análisis de datos, la decisión de publicar ni su redacción.

## Declaración.

Las opiniones expresadas en este manuscrito son únicamente responsabilidad de los autores y no reflejan necesariamente los criterios ni la política de la *RPSP/PAJPH* y / o de la OPS.
